# Anodic Dissolution Characteristics of GH4169 Alloy in NaNO_3_ Solutions by Roll-Print Mask Electrochemical Machining Using the Linear Cathode

**DOI:** 10.3390/ma17112729

**Published:** 2024-06-04

**Authors:** Ge Qin, Shiwei Li, Lei Han, Huan Liu, Shen Niu, Pingmei Ming, Liang Yan

**Affiliations:** School of Mechanical and Power Engineering, Henan Polytechnic University, Jiaozuo 454000, China; lswmzsx@163.com (S.L.); hanlei9854@163.com (L.H.); ns2019@hpu.edu.cn (S.N.); mingpingmei@163.com (P.M.); yanliang@hpu.edu.cn (L.Y.)

**Keywords:** GH4169 alloy, through-mask electrochemical micromachining (TMEMM), process parameter optimization, micro-pit structure forming

## Abstract

GH4169 alloy/Inconel 718 is extensively utilized in aerospace manufacturing due to its excellent high temperature mechanical properties. Micro-structuring on the workpiece surface can enhance its properties further. Through-mask electrochemical micromachining (TMEMM) is a promising and potential processing method for nickel-based superalloys. It can effectively solve the problem that traditional processing methods are difficult to achieve large-scale, high-precision and efficiency processing of surface micro-structure. This study explores the feasibility of electrochemical machining (ECM) for GH4169 using roll-print mask electrochemical machining with a linear cathode. Electrochemical dissolution characteristics of GH4169 alloy were analyzed in various electrolyte solutions and concentrations. Key parameters including cathode sizes, applied voltage and corrosion time were studied in the roll-print mask electrochemical machining. A qualitative model for micro-pit formation on GH4169 was established. Optimal parameters were determined through experiments: 300 μm mask hole and cathode size, 10 wt% NaNO_3_ electrolyte, 12 V voltage, 6 s corrosion time. The results demonstrate that the micro-pits with a diameter of 402.3 μm, depth of 92.8 μm and etch factor (EF) of 1.81 show an excellent profile and localization.

## 1. Introduction

Due to the rapid development of aerospace manufacturing and nanotechnology, the demand for high temperature resistant metamaterials is also increasing [1,2]. In order to achieve the significant improvement in the performance of gas turbines for space shuttles and rocket engines [3], high temperature resistant materials have been applied, widely represented by GH4169 alloy. GH4169 is a nickel-based alloy strengthened by homogeneous precipitation of γ′ and γ″ phases, and the γ″ phase plays a leading role in strengthening [4]. The existence of γ′ phase and γ″ phase can improve the high temperature strength, deformation resistance and fatigue resistance of GH4169 alloy by strengthening mechanism, including dispersion strengthening and dislocation obstruction, so as to enhance the high temperature mechanical properties of GH4169 alloy. It has been reported that GH4169 alloy can maintain high strength, and good stability and weldability at ultra-high temperature [5], which can effectively improve the high temperature resistance of gas turbines, especially at the inlet, so that its performance has also been greatly improved.

GH4169 alloy has mechanical properties of high hardness and yield strength, which makes the micro-structure processing have the disadvantages of serious tool wear, uneven processing surface and residual stress. Therefore, the traditional processing technology is difficult to meet its high efficiency and quality processing. Many researchers have studied its machining process by adjusting the processing method or adopting a new process. Zhu et al. [6] studied the effects of amplitude, frequency, spindle speed and feed speed on drilling force, processing quality and drill wear in the process of ultrasonic assisted drilling of nickel-based superalloy, and successfully processed high-quality micropores on its surface. Ma et al. [7] deeply studied the influence of geometric parameters of micro-milling tools on the machinability of Inconel 718. They reported that the geometric parameters of the machining tool can significantly affect the machining process of Inconel 718, and when the small rake angle tool was used for high-speed machining of Inconel 718, the machining wear of the machining tool can be effectively reduced, and the service life of the machining tool can be significantly improved. Chen et al. [8] designed a polycrystalline diamond tool (PCD) micro-end milling cutter, carried out three-dimensional simulation analysis and experimental verification, and successfully processed high-precision micro-groove structures on the surface of nickel-based superalloys through such tools. On the other hand, electrical discharge machining (EDM), as a non-contact special processing technology, is widely used in the processing and manufacturing of high hardness and strength materials. However, EDM also has defects that cannot be eradicated, such as edge fracture, burr and surface recast layer. Sun et al. [9] proposed and studied the discharge drilling method using the spiral micro-electrode, and completed the processing and preparation of high-precision micro-porous structures on the surface of nickel-based alloy. In addition, Zhang et al. [10] proposed a laser processing with high-temperature chemical etching method to complete the high-quality surface micro-porous structures processing of Inconel 718 under different processing media. However, due to the non-directionality and complexity of the combination of laser and chemical reaction, the honeycomb structure was generated during the processing.

Electrochemical machining (ECM) is an anodic electrochemical dissolution reaction process on a metal surface to realize the processing of surface micro-structure arrays [11,12]. This process has many advantages, such as high efficiency, no recast layer and tool wear, no residual stress, high material removal rate and is not affected by the mechanical properties of materials [13,14]. At the same time, compared with the traditional and other new processing methods of GH4169 alloy, ECM can use self-designed and manufactured machine tools or devices, without the need to separately purchase expensive machine tools or special equipment, such as high-precision lathes and laser equipment. The cost of operation and later maintenance is also low, and the application and promotion prospects are very broad. Ye et al. [15] used tube electrodes with different oblique angles to generate pulsating flow field in the electrochemical processing area, so that a micro-groove with a depth of 5 mm was stably machined on the surface of GH4169 alloy at one time. Liu et al. [16] established the electric field and flow field models of electrochemical milling by using the spiral micro-electrode, analyzed the influence of flow field and electric field on micro-structure forming, and successfully processed a series of complex micro-structures on the surface of Inconel 718. Huang et al. [17] successfully processed high-precision micro-structures on the surface of nickel-based alloy on ECM of nanosecond pulse. Through-mask electrochemical micromachining (TMEMM) is one of the variants of ECM, which is a machining method that uses an insulating material with a customized pattern on the surface as a mask to attach to the surface of the anode workpiece to limit its processing area [18,19,20]. It has the advantages of cold processing, flexible solvent, no tool loss and mass manufacturing of TMEMM, which can effectively alleviate the problems of serious stray corrosion and poor localization in the process of conventional electrochemical machining methods.

The roll-print mask electrochemical machining using the linear cathode is a technical method for electrochemical dissolution process using a rolling device with a linear cathode and an insulation mask. This has the advantages of high processing efficiency, no tool cathode loss, large-scale and high-efficiency continuous processing for the surface micro-structures [21,22]. The aim is to obtain the optimal process parameters for high precision, high efficiency and good localization of surface micro-structure arrays in the roll-print mask electrochemical machining using the linear cathode of GH4169 alloy in this paper. Therefore, the electrochemical dissolution characteristics of GH4169 alloy is firstly analyzed under static conditions of TMEMM. The polarization curves of GH4169 alloy in different solutions and different concentrations of NaNO_3_ electrolyte and the current efficiency in the process are obtained to determine the appropriate electrolyte processing parameters. The effects of main process parameters are clarified in detail on the machining performance of the workpiece, such as the linear cathode size, applied voltage and corrosion time. Meanwhile, the optimal process parameters are selected. Finally, on the basis of the optimal process parameters, the qualitative model of the surface micro-pit structure forming process of GH4169 alloy in NaNO_3_ electrolyte is established under the roll-print mask electrochemical machining using the linear cathode.

## 2. Experimental

### 2.1. GH4169 Alloy

The anode workpiece material applied in all experiments is GH4169 alloy. GH4169 is one of the precipitation-strengthened nickel-based alloys of Chinese Standard, which has similar micro-structure, mechanical and material properties with Inconel 718 of American Standard and NC19FeNb of French Standard [23,24,25,26]. Some characteristics of GH4169, Inconel 718 and NC19FeNb alloys are shown in Table 1. The chemical composition of GH4169 alloy is shown in Table 2. Cube-shaped GH4169 alloy specimens were prepared for electrochemical tests, and TMEMM experiments using a self-developed device to complete the preparation of surface micro-structures.

### 2.2. Basic Theory of the Roll-Print Mask Electrochemical Machining Using the Linear Cathode

**Figure 1:**
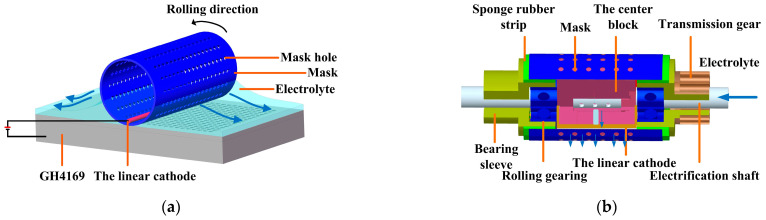
Principle of the roll-print mask electrochemical machining using the linear cathode. (**a**) Schematic diagram of the working process; (**b**) the rolling device.

The roll-print mask electrochemical machining makes a metal wire as the linear cathode using a rolling device, on which a mask and the linear cathode are installed (as shown in Figure 1).

During machining, the mask rotates along with the rolling device, while the position of the linear cathode remains stationary, selectively removing the workpiece material in the electrochemical processing area. Compared with conventional electrochemical machining, the small size linear cathode is used to limit the electric field below it. This makes the current density mainly concentrated in the electrochemical processing area to obtain better micro-structure etching morphology and geometric size profile.

### 2.3. Polarization Curves

A three-electrode configuration system was usually used to measuring electrochemical polarization curves of GH4169 alloy, which can effectively eliminate the voltage drop caused by the internal resistance of the solution and the polarization generated by the auxiliary electrode and can better realize the measurement and control of the potential. In this study, the electrochemical workstation was used to measure the electrochemical polarization properties of a GH4169 alloy workpiece in different electrolytes with different concentrations, and a platinum electrode and a saturated calomel electrode was used to be as the auxiliary electrode and the reference electrode, respectively. The scanning voltage range was −1.5~3 V, and the scanning speed was 10 mV/s.

### 2.4. Current Efficiency Measurement

Current efficiency is an important parameter to estimate the electrolytic erosion ability of materials. Therefore, in order to study the actual material removal rate of GH4169 alloy, the current efficiency of it under static conditions of TMEMM was measured by weighing method using the self-developed experimental device. In order to ensure the uniform and constant current distribution, the electrolytic power supply in the constant current mode was selected to precisely control the corrosion time of each detection.

During the measurement process, the rolling device and the anode workpiece remained stationary. Before the GH4169 alloy workpieces were weighed, they were cleaned by ultrasonic vibration and dried. The dissolution weight of the workpiece was recorded in each test. The current efficiency was determined by the actual anode dissolution mass, M, and the theoretical anode dissolution mass, m. The current efficiency of GH4169 alloy workpiece can be calculated by the following formula [27]:

η = M/m = M/ωρIt(1)

ηω = M/ρIt(2)

### 2.5. TMEMM Experiments

**Figure 2:**
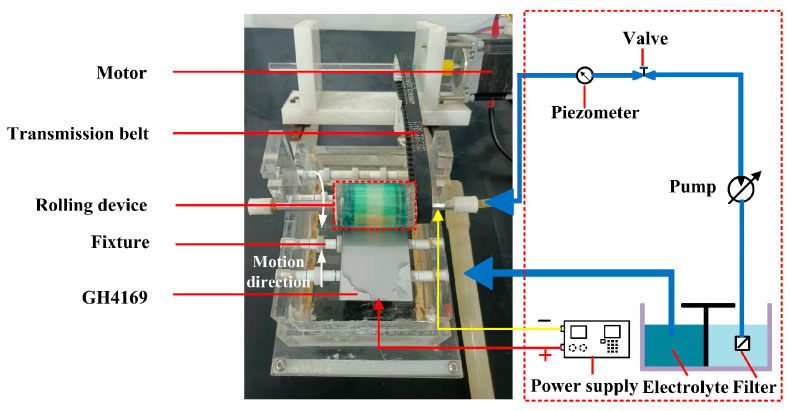
Schematic diagram of the experimental system of the roll-print mask electrochemical machining using the linear cathode.

The experiments of TMEMM were carried out to complete the machining of the surface micro-pits of GH4169 alloy using the self-developed experimental device (as shown in Figure 2).

The GH4169 alloy workpiece with a size of 10 mm × 10 mm × 10 mm was selected as the anode, and the linear copper wire was as the cathode. Polyimide (PI) with 0.1 mm thickness was used as a flexible mask material, and its surface was machined to customized micro-porous arrays by femtosecond laser process. Table 3 summarizes the process parameters of these experiments.

After experiments, the three-dimensional morphology and actual size of the micro-pit structures on the surface of GH4169 alloy can be observed by Olympus microscope (Olympus LEXT OLS5000, Tokyo, Japan). The micro-pit morphology on the surface of GH4169 alloy can be observed by a scanning electron microscope (SEM, Carl Zeiss NTS GmbH, Oberkochen, Germany).

The experiments were conducted by using the present experimental device to investigate the dissolution characteristics, the effects of the linear cathode size, applied voltage and corrosion time on the micro-pit etching morphology and geometric size profile of TMEMM on GH4169 alloy. The EF was measured to evaluate the electrochemical machining performance and machining accuracy of GH4169 alloy.

## 3. Results and Discussion

### 3.1. Electrochemical Characterization of GH4169 Alloy

The electrochemical dissolution characteristics of GH4169 alloy is important to explore its electrochemical machining performance. Therefore, the electrochemical polarization behavior of the GH4169 alloy workpiece was analyzed in different kinds of solutions and the current efficiency under the condition of TMEMM using the linear cathode.

**Figure 3:**
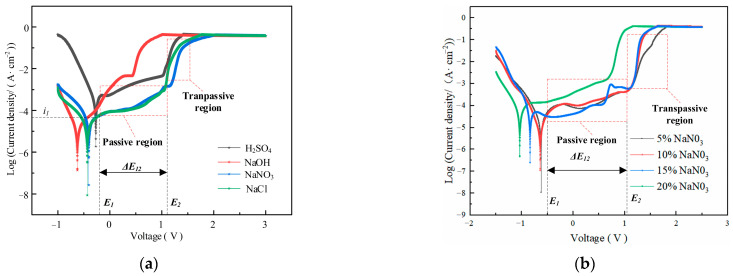
Determination of polarization curves in (**a**) Different electrolytes; (**b**) NaNO_3_ electrolyte with different concentration.

Figure 3 shows the influence of electrolyte composition and concentration on the electrochemical dissolution characteristics of GH4169 alloy.

The polarization curves of GH4169 alloy in the selected H_2_SO_4_, NaCl and NaNO_3_ solutions have apparent passivation zones. However, it is difficult to find the passivation regions in the polarization curves measured in strongly corrosive NaOH solution (as shown in Figure 3a). By comparing and analyzing the polarization curves measured in 10 wt% H_2_SO_4_, NaOH, NaCl and NaNO_3_ solutions, it is shown that the 10 wt% NaNO_3_ solution has the longest passivation region compared with H_2_SO_4_, NaOH and NaCl solutions, and its over-passivation zone is smooth and stable. Although the SO_4_^2−^ ions also possess passivating properties, the NO^3−^ ions exhibit stronger passivation, and the passivation film formed is more stable [28]. This is because the NO_3_^−^ ion has a strong passivation, and the passivation film formed is more stable. This indicates that the passivation film formed by the non-processing zone on the surface of GH4169 alloy in NaNO_3_ solution is more stable, the protective effect is stronger and the stray corrosion is less. Therefore, NaNO_3_ solution is selected to as the experiment solution of GH4169 alloy for TMEMM.

The corresponding polarization curves of GH4169 alloy in different concentrations of NaNO_3_ solution have obvious passivation zone and over-passivation zone, and the passivation zone does not change significantly with the increase in the concentration of NaNO_3_ solution (as shown in Figure 3b), which indicated that a stable and dense passivation film is easily formed on the surface of GH4169 alloy in NaNO_3_ solution and its compactness does not change significantly with the increase in the concentration. In the over-passivation zone, the surface of GH4169 alloy is severely dissolved, and the corresponding polarization curve is approximately coincident with Ohm’s law. The polarization curve slope indicates the difficulty of material removal of the workpiece. The larger the slope, the easier the material removal. For the polarization curve corresponding to 5 wt% NaNO_3_ solution, the slope is small after reaching the passivation region, which indicates that the dissolution efficiency is slow. Therefore, 10 wt% NaNO_3_ solution was chosen to account for both the accuracy and efficiency requirement of TMEMM on GH4169 alloy.

**Figure 4:**
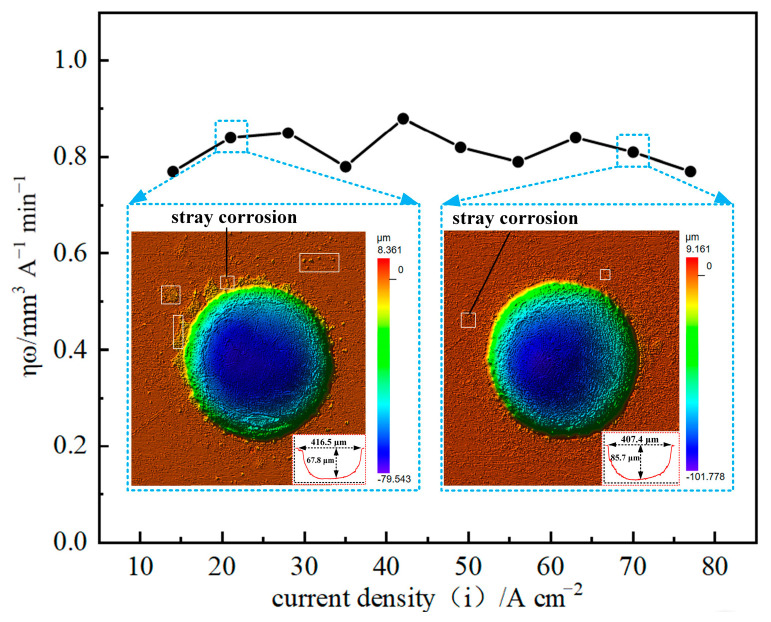
The ηω-j curve of GH4169 alloy in linear cathode electrolysis state.

The current efficiency of GH4169 alloy was measured under different current values on the electrochemical conditions of 10 wt% NaNO_3_ electrolyte, 1 L/min electrolyte flow rate and 6 s corrosion time using the linear cathode with a diameter of 0.3 mm and the mask hole with a diameter of 0.3 mm. As shown in Figure 4, the current efficiency of GH4169 alloy has no obvious change with the increase in current density.

When the current density is small, the current efficiency of GH4169 alloy is still high, but the micro-pit is shallow in depth (only 67.8 μm), which indicates that the stray corrosion in the non-processing area is serious at low current density, and the surface processing quality of GH4169 alloy is poor. With the increase in current density, the depth of micro-pit can reach 85.7 μm or even deeper, which indicates that the stray corrosion phenomenon in the non-processing area is gradually weakened, and the smooth machined surface can be obtained. Therefore, the moderate current density can be selected to ensure the processing efficiency and accuracy of the GH4169 alloy workpiece and the economy of processing in actual processing.

### 3.2. The Optimal Experimental Parameters of GH4169 Alloy under Static Conditions of TMEMM

The process parameters of electrochemical machining are an important factor affecting the surface accuracy of the workpiece. The process parameters including the linear cathode size, applied voltage and corrosion time were investigated. The EF was calculated to evaluate the processing localization under different conditions, which was used to select the optimal experimental parameters of GH4169 alloy.

#### 3.2.1. The Linear Cathode Size

**Figure 5:**
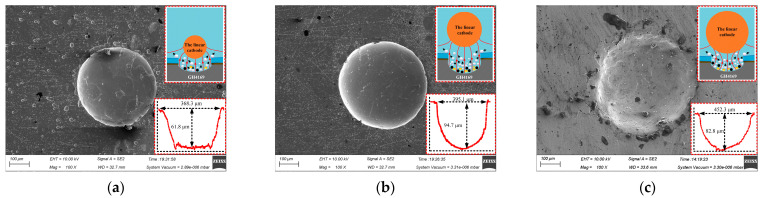
Effect of the linear cathode size on micro-pit forming. (**a**) d = 100 μm; (**b**) d = 300 μm; (**c**) d = 500 μm.

Figure 5 shows the micro-pit structure profile of the workpiece machined with different linear cathode diameter on the processing condition of 300 μm mask hole diameter, 6 s corrosion time, 12 V processing voltage.

When the linear cathode with a diameter of 100 μm was selected, the linear cathode size was smaller than the mask hole size and the applied electric field was mainly concentrated below the cathode. This was not uniform for the distribution of the processing area and the current density distribution at the bottom of the micro-pit, which resulted in uneven surface corrosion of the processed micro-pits and the appearance of some micro-cracks (as shown in Figure 5a). When the diameter of the linear cathode was 500 μm, the linear cathode size was larger than the size of the mask hole and the gap between the mask and the cathode was so narrow that the electrolyte was difficult to discharge, and the mass transfer of the flow field was poor. The electrolytic products produced by the processing accumulated at the bottom of the micro-pits, which led to poor forming quality of the micro-pits (as shown in Figure 5c). When the diameter of the linear cathode was 300 μm and equal to the size of the mask hole, the applied electric field was evenly distributed in the whole electrochemical machining area, and the machined micro-pit profile was better, as shown in Figure 5b. Therefore, the copper wire with a diameter of 300 μm can be selected as the linear cathode in this study.

#### 3.2.2. The Applied Voltage

**Figure 6:**
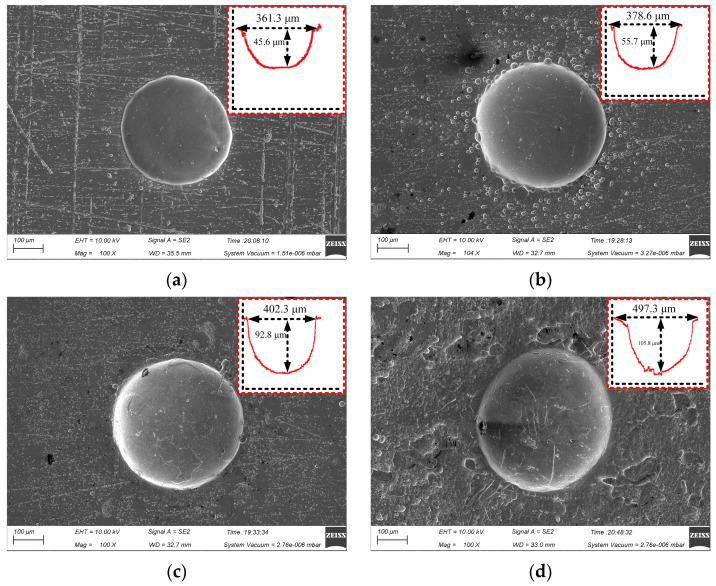
Effect of applied voltage on micro-pit forming. (**a**) U = 10 V; (**b**) U = 11 V; (**c**) U = 12 V; (**d**) U = 13 V.

Figure 6 shows the surface micro-pit morphology of GH4169 alloy of TMEMM using the linear cathode at different voltages (10, 11, 12 and 13 V).

When the applied voltage increased from 10 V to 13 V, the etching depth of the micro-pits increased from 45.6 μm to 105.8 μm. As shown in Figure 6c, when the applied voltage was 12 V, the diameter and depth of the micro-pit reached 402.3 μm and 92.8 μm, respectively. The bottom of the micro-pit was relatively smooth, and the stray corrosion in the non-processing area was significantly reduced, compared with the processed surface when the applied voltage was 11 V (as shown in Figure 6b). As the voltage increased, the diameter of the micro-pit increased significantly to 487.3 μm, but the micro-pit only increased to 105.8 μm. The stray corrosion in the non-processing area is more serious, as shown in Figure 6d at 13 V.

The reason for the above results is the complex composition of GH4169 alloy, the electrode potential of the phase with different element composition is different, and the order of dissolution is different. Therefore, when the applied voltage was small, the phase with more negative electrode potential in GH4169 alloy was electrochemically dissolved firstly, resulting in a large roundness deviation of the processed micro-pit. When the applied voltage reached 12 V, it exceeded the electrode potential of all phases and the etching speed of each composition tended to be consistent, so the micro-pit boundary was regular and the bottom was flat. When the applied voltage increased to 13 V or even higher, the electrochemical reaction was more intense. The electrolytic products cannot be discharged in time and accumulated at the bottom of the micro-pit. Meanwhile, the radial corrosion of the micro-pits was still ongoing, leading in poor processing quality of the micro-pit.

#### 3.2.3. The Corrosion Time

**Figure 7:**
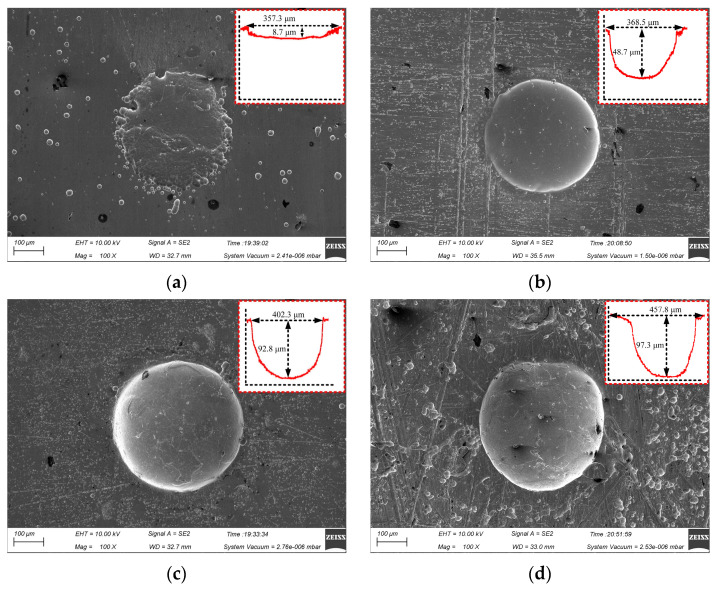
Effect of corrosion time on micro-pits forming. (**a**) t = 2 s; (**b**) t = 4 s; (**c**) t = 6 s; (**d**) t = 8 s.

Figure 7 shows the surface micro-pit morphology of GH4169 alloy under different corrosion time.

The actual electrochemical reaction time on the workpiece surface was too short to complete the breakdown of the passivation film on the surface of GH4169 alloy and the dissolution of the matrix material in the electrochemical processing area when the initial corrosion time was 2 s. The diameter of the machined micro-pit was 357.3 μm and the depth was only 8.7 μm, which had the irregular boundary and serious stray corrosion (as shown in Figure 7a). With the extension of the processing time, the stray corrosion was obviously weakened and the outline of the micro-pit was gradually formed, but the depth was still only 48.7 μm at 4 s, as shown in Figure 7b. When the processing time was increased to 6 s, the dissolution reaction of the GH4169 alloy was fully carried out, and the micro-pit with a diameter of 402.3 μm and a depth of 92.8 μm was processed. These micro-pits have clear boundary and regular shape, which indicated that there was no corrosion at the entrance of the micro-pits. When the corrosion time was further increased to 8 s, the diameter of the micro-pit increased to 457.8 μm, but the depth of the micro-pit was up from 92.8 μm to 97.3 μm due to the accumulation of electrolytic products. The aspect ratio decreased significantly.

The machining localization of the mask electrochemical machining process is usually described by the EF. The r_d_ is defined as the average etching rate, and its expression is as follows:

r_d_ = h⁄t (3)

When processing circular micro-structures on the surface of the workpiece, r_u_ is usually defined as the average lateral etching rate, and its expression is as follows:

r_u_ = u⁄t = (x_u_ − r_0_)⁄t (4)

EF = h⁄(x_u_ − r_0_) (5)

**Figure 8:**
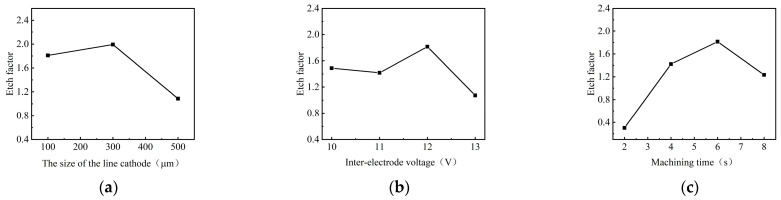
The effect of process parameters on the EF. (**a**) Size of the linear cathode; (**b**) applied voltage; (**c**) corrosion time.

Figure 8 shows the effect of process parameters on the EF of micro-pit on the GH4169 alloy surface.

As the size of the linear cathode increased, the EF increased first and then decreased, which indicates that the processing quality and localization are better using the linear cathode with the diameter of 300 μm (as shown in Figure 8a). This result is consistent with the result shown in Figure 5. With higher applied voltage, the EF remained basically unchanged at the beginning. When the applied voltage was 12 V, the EF was larger, which indicates that the processing quality and localization of the machined micro-pit are better (as shown in Figure 8b). With the progress of electrochemical reaction, the etch depth and the roundness of the micro-pits increased continuously, and the surface morphology and the localization of the micro-pits gradually became better when the corrosion time was 6 s (as shown in Figure 8c). The reason for this result is that the electrolytic products accumulated at the bottom of the micro-pit with the forming of the micro-pit and the excessive electrochemical reaction, which caused the aspect ratio, the EF, the processing quality and localization of the micro-pits to deteriorate (as shown in Figure 7).

### 3.3. Micro-Pit Forming Principle of GH4169 Alloy

**Figure 9:**
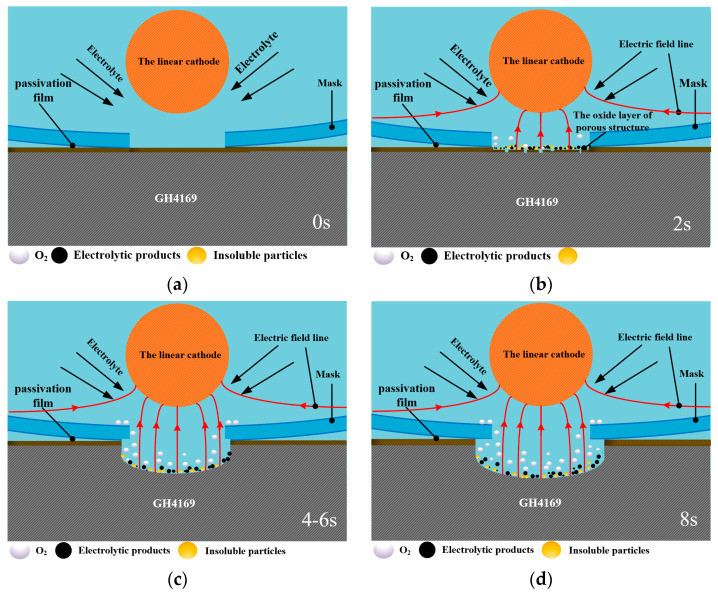
Schematic model of the electrochemical dissolution behavior of GH4169 alloy using the linear cathode in NaNO_3_ solution. (**a**) The initial workpiece surface; (**b**) The partial breakdown of surface passivation film; (**c**) The surface passivation film is completely broken down and the electrochemical reaction is basically completed.; (**d**) The electrochemical reaction is overdone.

Qualitative models of the micro-pit electrochemical dissolution of GH4169 alloy under the linear cathode electrochemical machining were proposed based on the above electrochemical characterization and machining experimental results. Figure 9 shows schematic diagrams of the micro-pit dissolution process of GH4169 alloy in NaNO_3_ solution.

According to the potentiodynamic polarization results (as shown in Figure 3), a thin passivating film is produced on the GH4169 alloy surface in NaNO_3_ solution when the electrochemical machining starts (as shown in Figure 9a). The passivating film gradually thins and is partially broken down via the electrochemical dissolution as shown in Figure 9b, and electrochemical pitting or crevice corrosion are initiated rapidly by local breakdowns of the passivating film that gradually exposes the GH4169 matrix material to the electrolyte. With the dissolution of the passivation film, the amount of the oxygen precipitation on the surface of the GH4169 alloy increases. Therefore, when the corrosion time is short, the electrolytic processing is mainly the dissolution of the passivation film and the matrix material is hardly electrochemical machined.

When the passivation film is completely broken down, violent electrochemical reactions occur on the surface of GH4169 alloy in the electrochemical machining zone, accompanied by the generation of black electrolytic products and insoluble particles (as shown in Figure 9c,d), and the metal material is dissolved downward and the micro-pit is gradually formed. With the increase in electrochemical reaction time of the micro-pit, the electrolytic product and the insoluble particles gradually increases, and the evolution amount of oxygen on the metal surface is also increased. Due to the small inter-electrode gap between the mask and the workpiece, the discharge speed of the electrolytic products is slow, and they will stay on the surface of the micro-pit, which will affect the further progress of electrochemical reaction and the quality of electrolytic surface. This result can also be seen in Figure 7.

**Figure 10:**
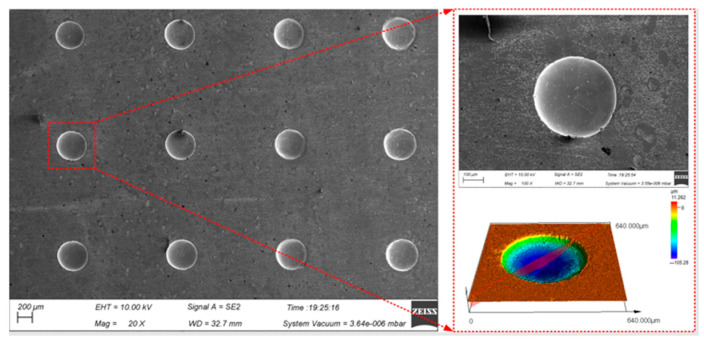
Surface topography of micro-pit arrays on the GH4169 alloy.

The continuous processing was carried out by using the optimal process parameters above on the presented device to realize the large-area processing of the micro-pit arrays on the surface of GH4169 alloy (as shown in Figure 10).

**Figure 11:**
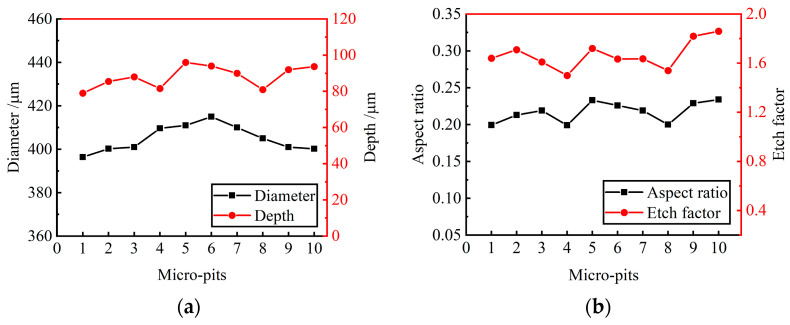
Size distribution of micro-pit arrays. (**a**) Diameter and depth of micro-pits; (**b**) aspect ratio and EF of micro-pits.

Ten micro-pits were randomly selected from the micro-pit arrays to measure their surface morphology. The micro-pits obtained by the presented method exhibited a relatively concentrated size distribution, with the average diameter of 405.85 ± 9.45 μm, the average depth of 87.5 ± 8.5 μm, the average aspect ratio of 0.22 and the average EF of 1.67 (as shown in Figure 11). The variation coefficient of the diameter and depth is 0.01 and 0.06, respectively. The results show that the micro-pit arrays have good localization and high uniformity.

**Figure 12:**
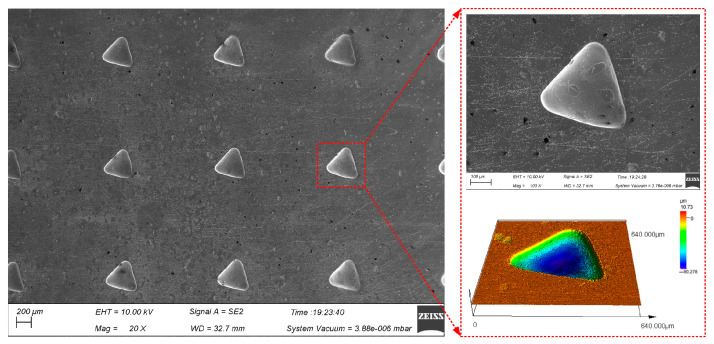
Surface topography of triangular micro-pit arrays on the GH4169 alloy.

Figure 12 shows triangular micro-pit arrays of GH4169 alloy machined under the above electrolytic processing conditions using an equilateral triangular pattern micro-porous arrays mask with a side length of 0.3 mm.

**Figure 13:**
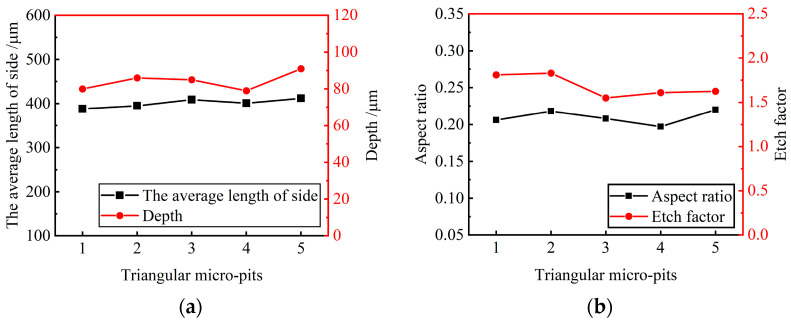
Size distribution of triangular micro-pit arrays. (**a**) Average length of side and depth of triangular micro-pits; (**b**) aspect ratio and EF of triangular micro-pits.

Five micro-pits were randomly selected to measure their surface morphology from the triangular micro-pit arrays. The size distribution is shown in Figure 13.

The average length of three sides and the average depth of the triangular micro-pits is 400.25 ± 12.05 μm and 85.4 ± 6.2 μm, respectively. The average aspect ratio is 0.21, the average EF is 1.69, indicating that the triangular micro-pit arrays have high uniformity and forming quality.

## 4. Conclusions

The effects of applied electrolyte composition and concentration, linear cathode size, voltage and corrosion time on the electrochemical machining performance of GH4169 alloy were studied experimentally using roll-print mask electrochemical machining with a linear cathode. Based on the experimental results, the following important conclusions can be drawn:

1. Compared with H_2_SO_4_, NaOH and NaCl solutions, the passivation film formed on the surface of the corrosion-prone GH4169 alloy is more stable due to the strong passivation of NO_3_^−^ ions in NaNO_3_ solution, which is more suitable for TMEMM of GH4169 alloy. There is no obvious change in trend of current efficiency with the change in current density in the process of the linear cathode TMEMM of GH4169 alloy;
2. When the size of the mask hole is equal to the size of the linear cathode under the same experiment conditions of electrochemical machining, it is most conducive to machining micro-pit structure with the clear outline on the surface of GH4169 alloy due to a uniformly distributed electric field. Meanwhile, moderate voltage and corrosion time are beneficial to improve the localization of micro-pits on GH4169 alloy surface;
3. Considering the effect of process parameters on the electrochemical processing performance, the optimal process parameters of mask hole with 300 μm diameter, 10% NaNO_3_, 12V voltage and 6 s corrosion time are selected. The processing results show that the micro-pits with a diameter of 402.3 μm, a depth of 92.8 μm and an EF of 1.81 can be obtained by using the above process parameters. These results show good localization, surface morphology and processing performance.
4. Based on the above optimized micro-pit processing conditions and parameters, high-precision, localized and uniform micro-pit and equilateral triangular arrays can be processed on the surface of GH4169 alloy. The diameter of the micro-pit arrays is 405.85 ± 9.45 μm, the depth is 87.5 ± 8.5 μm, the average aspect ratio is 0.22 and the average EF is 1.67. The average side length of the equilateral triangular array is 400.25 ± 12.05 μm, the depth is 85.4 ± 6.2 μm, the average aspect ratio is 0.21 and the average EF is 1.69.
5. In future work, we can continue to optimize the relevant microstructure processing technology or explore new processing methods to continue to improve some surface properties of GH4169 alloy. In addition, the influence of surface micro-structures can be further studied on the mechanical properties of GH4169 alloy, such as fatigue resistance and tensile strength.

## Figures and Tables

**Table 1:** Some characteristics of GH4169, Inconel 718 and NC19FeNb alloys.

Characteristic	GH4169 Alloy	Inconel 718	NC19FeNb
Chemical Composition	Nickel, chromium, molybdenum, iron and titanium, mainly	Nickel, chromium, iron, cobalt and titanium, mainly	Nickel, chromium, molybdenum, iron and niobium, mainly
Intensity	High strength, high temperature resistance	High strength, high temperature resistance	Medium strength, high temperature resistance
Corrosion Resistance	Excellent corrosion resistance	Excellent corrosion resistance	Excellent corrosion resistance
Machinability	Good machinability	Good machinability	Good machinability
Application	Aerospace, petrochemical and other high-end fields	Aerospace, energy and other fields	Chemical, medical and other fields

**Table 2:** Chemical composition of GH4169 alloy/Inconel 718 (%).

Composition	C	Si	Mn	S	P	Ni	Cr	Al	Cu	Co	Ti	Mo	Nb	B	Ta	Fe
Percentage	0.038	0.16	0.12	0.001	0.01	52.75	17.96	0.55	0.022	0.018	1.09	3.05	5.13	0.002	0.01	Bal

**Table 3:** Main experimental conditions and parameters.

Parameter	Value
Electrolytic solution	H_2_SO_4_, NaOH, HCl, NaNO_3_
H_2_SO_4_, NaOH, HCl electrolyte	10 wt%
NaNO_3_ electrolyte	5 wt%, 10 wt%, 15 wt%, 20 wt%
The anode workpiece	GH4169 alloy
The linear cathode	0.1–0.5 mm(diameter), Cu
Inter-electrode gap	0.1 mm
Thickness of the mask	0.1 mm
Corrosion time	0–8 s
Applied voltage	10–13 V
Electrolyte flow rate	1 L/min

## Data Availability

Data are contained within the article.

## Abbreviation

η	current efficiency
M/g	the actual anodic dissolution mass
m/g	the theoretical anodic dissolution mass
ω/cm^3^·(A·s)^−1^	the material theoretical volume electrochemical equivalent
ρ/g·cm^−3^	the material density
I/A	current
t/s	the corrosion time
h/μm	the etching depth of micro-pit
u/μm	the lateral etching width
x_u_/μm	the etching radius
r_0_/μm	the mask hole radius

## References

[1] Lalegani Z., Seyyed Ebrahimi S.A., Hamawandi B., La Spada L., Batili H., Toprak M.S. (2022). Targeted dielectric coating of silver nanoparticles with silica to manipulate optical properties for metasurface applications. Mater. Chem. Phys..

[2] Lincoln R.L., Scarpa F., Ting V.P., Trask R.S. (2019). Multifunctional composites: A metamaterial perspective. Multifunct. Mater..

[3] Chittewar S.L., Patil N.G. (2021). Surface integrity of conventional and additively manufactured nickel superalloys: A review. Mater. Today Proc..

[4] Zhang M.H., Zhang B.C., Wen Y.J., Qu X.H. (2022). Research progress on selective laser melting processing for nickel-based superalloy. Int. J. Miner. Metall. Mater..

[5] Qiao Z., Li C., Zhang H.J., Liang H.Y., Liu Y.C., Zhang Y. (2020). Evaluation on elevated-temperature stability of modified 718-type alloys with varied phase configurations. Int. J. Miner. Metall. Mater..

[6] Zhu X.X., Wang W.H., Jiang R.S., Zhang Z.F., Huang B., Ma X.W. (2020). Research on ultrasonic-assisted drilling in micro-hole machining of the DD6 superalloy. Adv. Manuf..

[7] Ma J.W., Jia Z.Y., He G.Z., Liu Z., Zhao X.X., Qin F.Z. (2019). Influence of cutting tool geometrical parameters on tool wear in high-speed milling of Inconel 718 curved surface. Proc. Inst. Mech. Eng. Part B J. Eng. Manuf..

[8] Chen N., Yuan Y., Guo C., Zhang X.L., Hao X.Q., He N. (2020). Design, optimization and manufacturing of polycrystalline diamond micro-end-mill for micro-milling of GH4169. Diam. Relat. Mater..

[9] Gong S.Q., Sun Y., Jin L.Y., Su Z.P. (2020). Experimental study on fabricating micro-holes in DD5 single-crystal nickel-based superalloy using electrical discharge drilling. Arch. Civ. Mech. Eng..

[10] Zhang Q., Sun S.F., Zhang F.Y., Wang J., Lv Q.Q., Shao Y., Liu Q.Y., Shao J., Liu X.F., Zhang Y. (2020). A study on film hole drilling of IN718 superalloy via laser machining combined with high temperature chemical etching. Int. J. Adv. Manuf. Technol..

[11] Zhan S.D., Zhao Y.H. (2020). Plasma-assited Electrochemical machining of microtools and microstructures. Int. J. Mach. Tools Manuf..

[12] Wang J., Xu Z.Y., Wang J.T., Xu Z.L., Zhu D. (2021). Electrochemical machining of blisk channels with rotations of the bathodeand the workpiece. Int. J. Mech. Sci..

[13] Liu W.D., Ao S.S., Li Y., Liu Z.M., Wang Z.M., Luo Z., Wang Z.P., Song R.F. (2017). Jet electrochemical machining of TB6 titanium alloy. Int. J. Adv. Manuf. Technol..

[14] Spieser A., Ivanov A. (2015). Design of an electrochemical micromachining machine. Int. J. Adv. Manuf. Technol..

[15] Ye Z.S., Qiu G.L., Chen X.L. (2022). Electrochemical Milling of Deep-Narrow Grooves on GH4169 Alloy Using Tube Electrode with Wedged End Face. Micromachines.

[16] Liu Y., Xu X.D., Guo C.S., Kong H.H. (2019). Analysis on machining performance of nickel-base superalloy by electrochemical micro-milling with high-speed spiral electrode. Micromachines.

[17] Huang S.F., Liu Y. (2014). Electrochemical micromachining of complex shapes on nickel and nickel-based superalloys. Mater. Manuf. Process..

[18] Datta M. (1998). Microfabrication by electrochemical metal removal. IBM J. Res. Dev..

[19] Zhao R.C., Huang L., Zhao H.Y., Cao Y., Tian W.J., Wang N. (2022). Study of Mask Electrochemical Machining for Ring Narrow Groove under the Action of Multiple Physical Fields. Coatings.

[20] Li Z.L., Dai Y. (2022). Simulation Analysis and Process Evaluation of Cooling Hole Forming Precision in Mask Assisted Electrochemical Machining Based on GH4169. Materials.

[21] Qin G., Li M., Han L., Ming P.M., Niu S., Yan L., Zheng X.S., Zhang X.M., Li S.W. (2024). Electrochemical machining process for micropit arrays using a rolling device with a linear cathode and a soft mask. Int. J. Adv. Manuf. Technol..

[22] Qin G., Li M., Li Y.L., Ming P.M., Zhang X.M., Han L., Yan L., Zheng X.S., Niu S. (2023). Experimental Study of Electrolytic Machining Process for Micro-pit Array on Workpiece Surface Using Linear Cathode and Rolling Printing Mask. Surf. Technol..

[23] Hu D.Y., Mao J.X., Song J., Meng F.C., Shan X.M., Wang R.Q. (2016). Experimental investigation of grain size effect on fatigue crack growth rate in turbine disc superalloy GH4169 under different temperatures. Mater. Sci. Eng. A.

[24] Deng G.J., Tu S.T., Zhang X.C., Wang Q.Q., Qin C.H. (2015). Grain size effect on the small fatigue crack initiation and growth mechanisms of nickel-based superalloy GH4169. Eng. Fract. Mech..

[25] Akhtar W., Sun J.F., Chen W.Y. (2016). Effect of machining parameters on surface integrity in highspeed milling of super alloy GH4169/Inconel 718. Mater. Manuf. Process..

[26] Niu S., Qu N.S., Fu S.X., Fang X.L., Li H.S. (2017). Investigation of inner-jet electrochemical milling of nickel-based alloy GH4169/Inconel 718. Int. J. Adv. Manuf. Technol..

[27] Liu W.D., Luo Z., Kunieda M. (2020). Electrolyte jet machining of Ti1023 titanium alloy using NaCl ethylene glycol-based electrolyte. J. Mater. Process. Technol..

[28] Niu S., Yu C.Y., Ming P.M., Wang S.R., Li S.S., Qin G. (2024). Experimental study on jet electrochemical micromilling of Ti-6Al-4V based on NaCl solution. Aeronaut. Manuf. Technol..

